# Therapeutic Effects of DNase I on Peripheral and Local Markers of Liver Injury and Neutrophil Extracellular Traps in a Model of Alcohol-Related Liver Disease

**DOI:** 10.3390/ijms26051893

**Published:** 2025-02-22

**Authors:** Paulína Belvončíková, Andrej Feješ, Barbora Gromová, Ľubica Janovičová, Anna Farkašová, Pavel Babál, Roman Gardlík

**Affiliations:** 1 Institute of Molecular Biomedicine, Faculty of Medicine, Comenius University, Sasinkova 4, 811 08 Bratislava, Slovakia; belvoncikova2@uniba.sk (P.B.); andrej.fejes@fmed.uniba.sk (A.F.); gromova10@uniba.sk (B.G.); lubica.janovicova@fmed.uniba.sk (Ľ.J.); farkasova140@uniba.sk (A.F.); 2 Institute of Pathological Anatomy, Faculty of Medicine, Comenius University, Sasinkova 4, 811 08 Bratislava, Slovakia; pavel.babal@fmed.uniba.sk

**Keywords:** alcohol-related liver disease, neutrophil extracellular traps, cell-free DNA, deoxyribonuclease

## Abstract

Alcohol-related liver disease (ALD) is a leading cause of chronic liver conditions globally. Chronic alcohol consumption induces liver damage through various mechanisms, including neutrophil extracellular trap (NET) formation. Extracellular DNA (ecDNA), released from damaged hepatocytes and NETotic neutrophils, has emerged as a potential biomarker and contributor to liver disease pathology. Enzyme DNases could be an effective therapy for the denaturation of immunogenic ecDNA. This study investigated the circulating ecDNA and NET markers in ALD and therapeutic effect of DNase I in a murine model of ALD. Female C57BL/6J mice were fed a control diet (n = 13) or Lieber–DeCarli ethanol diet for 10 days followed by a binge ethanol dose to mimic acute-on-chronic alcoholic liver injury. From day 5, mice fed ethanol were randomized into an ethanol diet group (n = 17) and ethanol + DNase group (n = 5), which received additional DNase I treatment every 12 h. Liver damage markers were analyzed. Circulating ecDNA and NETosis were measured by fluorometry and cytometry, respectively. DNase I activity was analyzed with single radial enzyme dispersion assay. The ethanol-fed mice exhibited increased mortality, neutrophil infiltration and structural damage in the liver. Total circulating ecDNA levels and NET markers did not differ between groups. DNase activity was higher in ethanol-fed mice compared to controls and additional daily administration of DNase prevented liver injury. These findings suggest that alcohol-induced liver injury modestly influences systemic NETosis and ecDNA levels. However, increased DNase activity can prevent disease progression and enhanced systemic degradation of ecDNA using DNase I.

## 1. Introduction

Alcohol consumption is one of the major causes of chronic liver diseases worldwide, with an estimated global prevalence of alcohol-related liver disease (ALD) of about 4.8% [1]. Extensive and chronic alcohol consumption leads to steatosis and alcohol-associated steatohepatitis (ASH) and may progress to cirrhosis and hepatocellular carcinoma [2]. Recognizing its profound global impact on public health, research efforts are increasingly directed toward understanding the pathogenesis of ALD.

Ethanol oxidation occurs primarily in the liver by metabolizing it to acetaldehyde and acetate through dehydrogenase enzymes. Ethanol itself, or reactive products of its metabolism such as acetaldehyde, increases oxidative stress and promotes reactive oxygen species (ROS) formation in the liver [3]. Increased oxidative stress directly damages mitochondria, leading to hepatocyte apoptosis or necrosis and the release of DNA of mitochondrial (mtDNA) or nuclear (ncDNA) origin into extracellular space [4,5]. Simultaneously, increased oxidative stress stimulates the immune system, e.g., by activating immune cells and promoting cytokine production [6]. Neutrophils represent the immune system’s first response to sterile or bacterial stimuli. Their activation can induce several defense mechanisms, including ROS generation, degranulation, phagocytosis, or the formation of neutrophil extracellular traps (NETs). NETs represent web-like structures composed of DNA and nuclear, granular, and cytosolic proteins which are expelled from neutrophils in a process of specific cell death called NETosis. During NETosis, DNA reaches the extracellular space where it can serve as a damage-associated molecular pattern (DAMP), triggering the immune system pathways [7,8,9]. Collectively, DNA physiologically resides within the nucleus and mitochondria. However, when expelled outside the cells, either from damaged hepatocytes or by NETosis, it may serve as a DAMP, repeatedly activate neutrophils, promote ROS and NETs formation, multiply pro-inflammatory signaling pathways and finally contribute to the progression of tissue damage [6,7,8].

A recent study showed that neutrophils from ASH patients show impairment in antibacterial activities and display increased ROS production as compared to controls [10]. Circulating ecDNA and increased NET formation serve as critical factors in the pathology of liver diseases [11]. Increased ecDNA concentrations were reported in patients with non-alcoholic steatosis and were linked to the disease’s severity [11]. Additionally, studies suggest that circulating ecDNA may not only be a biomarker of metabolic dysfunction-associated steatotic liver disease (MASLD) but also an active player in ALD and metabolic dysfunction-associated steatohepatitis (MASH) through enhanced NET formation. This ecDNA can originate from the damaged hepatocytes or already NETotic neutrophils [12,13]. Circulating ecDNA and NETs could thus emerge as promising early markers and possibly also therapeutic targets in ALD.

One potential strategy for reducing the immunogenicity of self-DNA is to employ deoxyribonuclease (DNase) enzymes, which are capable of cleaving phosphodiester bonds. Several studies have shown the high potential of DNase therapy in a variety of diseases, where evidence suggests the involvement of DNA-related DAMPs in the progression of sterile inflammation [14,15,16].

To study the role of circulating ecDNA and NET formation in ALD, the animal model of chronic alcohol feeding with additional ethanol binge dose could be used [17,18].

Collectively, this study aims to investigate the role of ecDNA and NET formation in the progression of ALD, using a mouse model of chronic-plus-binge ethanol feeding that mimics acute-on-chronic ALD, and, in addition, to evaluate the effect of DNase I therapy.

## 2. Results

**Figure 1:**
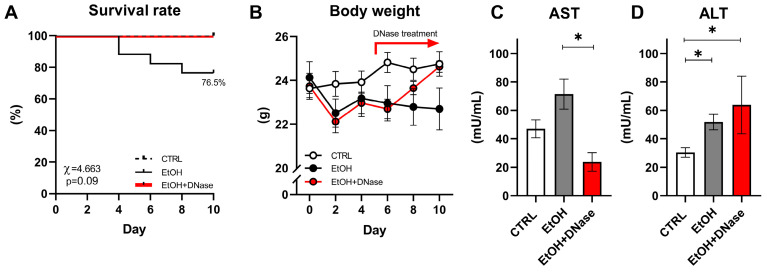
The course of the disease and liver-damage-associated markers: (**A**) survival rate; (**B**) dynamics of body weight; (**C**) aspartate aminotransferase (AST) activity in plasma; (**D**) alanine aminotransferase (ALT) activity in plasma. Data are presented as mean ± SEM; * *p* < 0.05; CTRL—control group (n = 13); EtOH—experimental ALD group (n = 13); EtOH + DNase—experimental ALD group with DNase I treatment (n = 5).

We observed approx. 23.5% mortality in the EtOH group starting from the 4th to 8th day of the experiment, while no mice from the CTRL and EtOH + DNase I-treated groups died (χ = 4.663; *p* = 0.09; Figure 1A). Only alive mice were included in the rest of the data analysis. The body weight of the CTRL, EtOH and EtOH + DNase groups changed over the 10 days of the experiment but with no difference between groups (group: *p* = ns; time: *p* < 0.001; interaction: *p* < 0.001; Figure 1B). Moreover, chronic-plus-binge ethanol feeding changed plasma AST (F(2,28) = 5.245, *p* < 0.05) and ALT (F(2,28) = 7.868, *p* < 0.01) activities (Figure 1C,D). Specifically, according to the unpaired *T*-test, the EtOH + DNase group showed lower AST activity (*p* < 0.05; Figure 1C) compared to the EtOH group, but increased ALT activity compared to the EtOH group (*p* < 0.05; Figure 1D) and control group (*p* < 0.05; Figure 1D).

**Figure 2:**
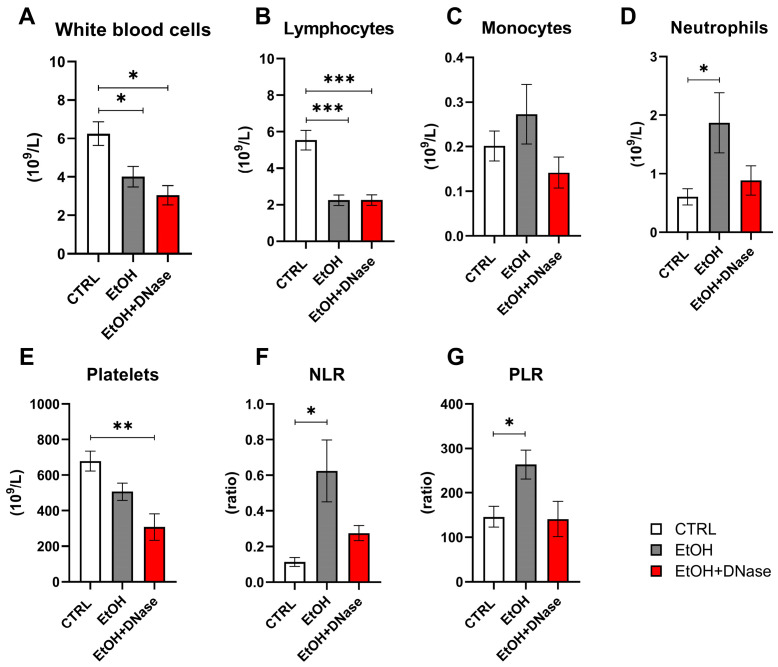
Blood cell count: (**A**) white blood cell count; (**B**) lymphocyte count; (**C**) monocyte count; (**D**) neutrophil count; (**E**) platelet count; (**F**) neutrophil-to-lymphocyte ratio; (**G**) platelet-to-lymphocyte ratio. Data are presented as mean ± SEM; * *p* < 0.05, ** *p* < 0.01, *** *p* < 0.001; CTRL—control group (n = 13); EtOH—experimental ALD group (n = 13); EtOH + DNase—experimental ALD group with DNase I treatment (n = 5).

The counts of white blood cells and lymphocytes in EtOH and EtOH + DNase groups were lower than in the control group (white blood cells: F(2,28) = 6.47, *p* < 0.05; Figure 2A; lymphocytes: F(2,27) = 18.39, *p* < 0.001; Figure 2B). Monocyte counts did not differ between ethanol groups (EtOH, EtOH + DNase) and the control group (F(2,27) = 1.16, *p* = ns; Figure 2C). The neutrophil counts were the highest in the EtOH group compared to the EtOH + DNase group and control group (F(2,28) = 3.36), *p* < 0.05; Figure 2D). On the contrary, the platelet count was lower in the ethanol groups (EtOH, EtOH + DNase) than in the control group (F(2,28) = 7.60, *p* < 0.01; Figure 2E). The neutrophil-to-lymphocyte (NLR) ratio was higher in the animals that received ethanol but not in the animals that received ethanol and DNase I treatment compared to control mice (F(2,27) = 4.84, *p* < 0.05; Figure 2F). Moreover, the platelet-to-lymphocyte (PLR) ratio was also higher in the ethanol group compared to control mice but not in the mice that received ethanol and DNase I treatment (F(2,27) = 5.36, *p* < 0.05; Figure 2G).

**Figure 3:**
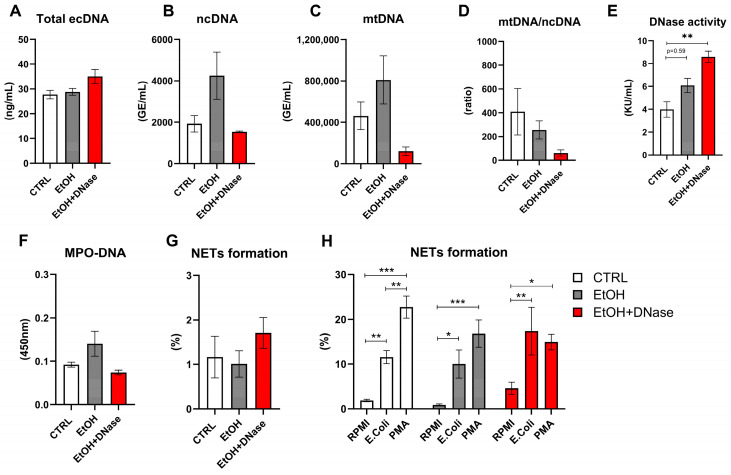
NETs-associated peripheral markers: (**A**) concentration of extracellular DNA (ecDNA); (**B**) concentration of nuclear DNA (ncDNA); (**C**) concentration of mitochondrial DNA (mtDNA); (**D**) mtDNA/ncDNA ratio; (**E**) DNase activity; (**F**) myeloperoxidase–DNA complex count (MPO-DNA); (**G**) percentage of in vivo NET formation; (**H**) percentage of ex vivo NET formation induced by different stimuli: RPMI, *Escherichia coli* (*E. coli*) and PMA. Data are presented as mean ± SEM; * *p* < 0.05, ** *p* < 0.01, *** *p* < 0.001; CTRL—control group (n = 13); EtOH—experimental ALD group (n = 13); EtOH + DNase—experimental ALD group with DNase I treatment (n = 5).

The concentration of circulating ecDNA did not differ between both ethanol groups and control mice (F(2,27) = 3.10, *p* = ns; Figure 3A). Similarly, no differences were observed between groups in the concentration of ncDNA (F(2,24) = 3.17; *p* = ns; Figure 3B), as well as in the mtDNA concentration (F(2,27) = 2.47, *p* = ns; Figure 3C). The mitochondrial fraction and ratio of mitochondrial and nuclear fractions did not differ between the two ethanol groups and the control group (F(2,24) = 1.06, *p* = ns; Figure 3D). DNase activity in plasma was higher in the ethanol mice treated with DNase I compared to the control group (F(2,26) = 7.60; *p* < 0.01; Figure 3E) but the ethanol without DNase I treatment group had a trend of higher DNase activity (F(2,26) = 7.60; *p* = 0.06). The MPO-DNA complex, as well as the percentage of in vivo NETotic neutrophils in circulation, did not differ between the EtOH, EtOH + DNase-treated mice and control mice (MPO-DNA complex: F(2,28) = 2.37, *p* = ns, Figure 3F; NET formation: F(2,24) = 0.67, *p* = ns, Figure 3G). The NET formation ex vivo varies according to the type of stimuli and group (group: F(2,39) = 1.34, *p* = ns; stimuli: F(2,39) = 30,67, *p* < 0.001; interaction: F(4,39) = 2,16, *p* = ns, Figure 3H). Neutrophils from mice that received ethanol showed a higher ability to release NETs under the *E. coli* and PMA stimuli than RMPI (control) (EtOH group: *E. coli* vs. RPMI: *p* < 0.05; PMA vs. RPMI: *p* < 0.001; Figure 3H). Interestingly, neutrophils from ethanol-fed mice released NETs in the same manner under the PMA and *E. coli* stimuli (*p* = ns; Figure 3H). A similar capacity to form NETs was observed in mice that received ethanol and DNase I treatment (EtOH + DNase: *E. coli* vs. RPMI: *p* < 0.01; PMA vs. RPMI: *p* < 0.05; *E. coli* vs PMA: *p* = ns; Figure 3H). Moreover, neutrophil from control mice showed a higher release of NETs in the presence of *E. coli* and PMA compared to RPMI control stimulus (control group: *E. coli* vs. RPMI: *p* < 0.01; PMA vs. RPMI: *p* < 0.001; Figure 3H). More importantly, as expected, neutrophils from control mice showed higher NET production in the presence of PMA than *E. coli* (*p* < 0.01; Figure 3H). No differences were found between experimental groups in any of the tested stimuli.

**Figure 4:**
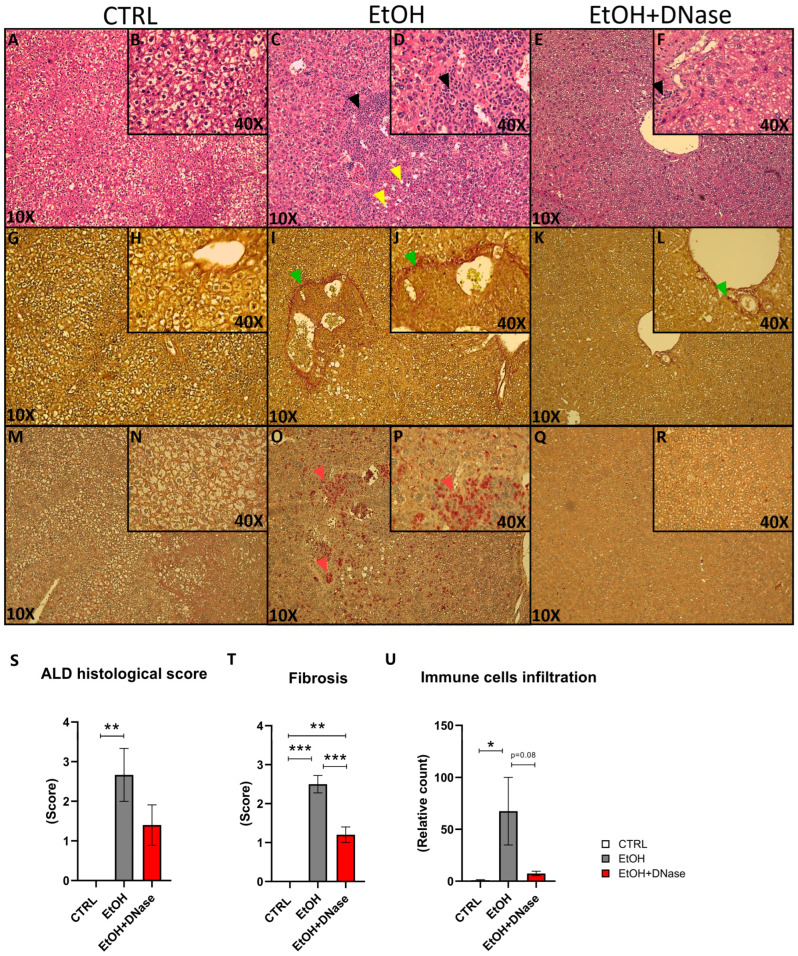
Representative pictures of liver histology and histology score: (**A**,**B**) hematoxylin and eosin staining (H&E) in the CTRL group; (**C**,**D**) H&E in the EtOH group; (**E**,**F**) H&E in the EtOH + DNase group; (**G**,**H**) Van Gieson staining in the CTRL group; (**I**,**J**) Van Gieson staining in the EtOH group; (**K**,**L**) Van Gieson staining in the EtOH + DNase group; (**M**,**N**) naphthol-AS-D-chloroacetate esterase staining in the CTRL group; (**O**,**P**) naphthol-AS-D-chloroacetate esterase staining in the EtOH group; (**Q**,**R**) naphthol-AS-D-chloroacetate esterase staining in the EtOH + DNase group; (**S**) ALD histological score; (**T**) liver fibrosis score; (**U**) immune cell infiltration in the liver. H&E staining: black arrow represents lobular inflammation; yellow arrow represents steatosis. Van Gieson staining: green arrow represents fibrosis; naphthol-AS-D-chloroacetate esterase staining. Red arrow represents immune cell infiltration. Data are presented as mean ± SEM; * *p* < 0.05, ** *p* < 0.01, *** *p* < 0.001; CTRL—control group (n = 5); EtOH—experimental ALD group (n = 5); EtOH + DNase—experimental ALD group with DNase I treatment (n = 5).

Lastly, the H&E staining showed that chronic-plus-binge ethanol feeding resulted in mild steatosis but massive lobular infiltration of immune cells (Figure 4A–D). This was reduced after DNase I treatment (Figure 4E,F). Similarly, liver fibrosis was visible only in the EtOH mice and less in DNase-treated ones (Figure 4G–L). In addition, neutrophils infiltrated the liver only in the EtOH group (Figure 4M–R). In summary, ethanol feeding caused liver tissue damage (F(2,13) = 6.81; *p* < 0.01; Figure 4S), as shown by the total NASH score (represented by the sum of steatosis, ballooning cells and lobular infiltration). Van Gieson staining showed increased fibrosis (F(2,13) = 48.43; *p* < 0.001; Figure 4T). Compared to the control group, a significantly higher fibrosis was shown in EtOH mice (*p* < 0.001) and EtOH + DNase mice (*p* < 0.001). However, EtOH + DNase group had significantly lower fibrosis compared with the EtOH group (*p* < 0.01). Lastly, immune cells infiltrated the liver only in the EtOH mice, but not in DNase-treated ones (F(2,13) = 4.27; *p* < 0.05; Figure 4U).

## 3. Discussion

**Figure 5:**
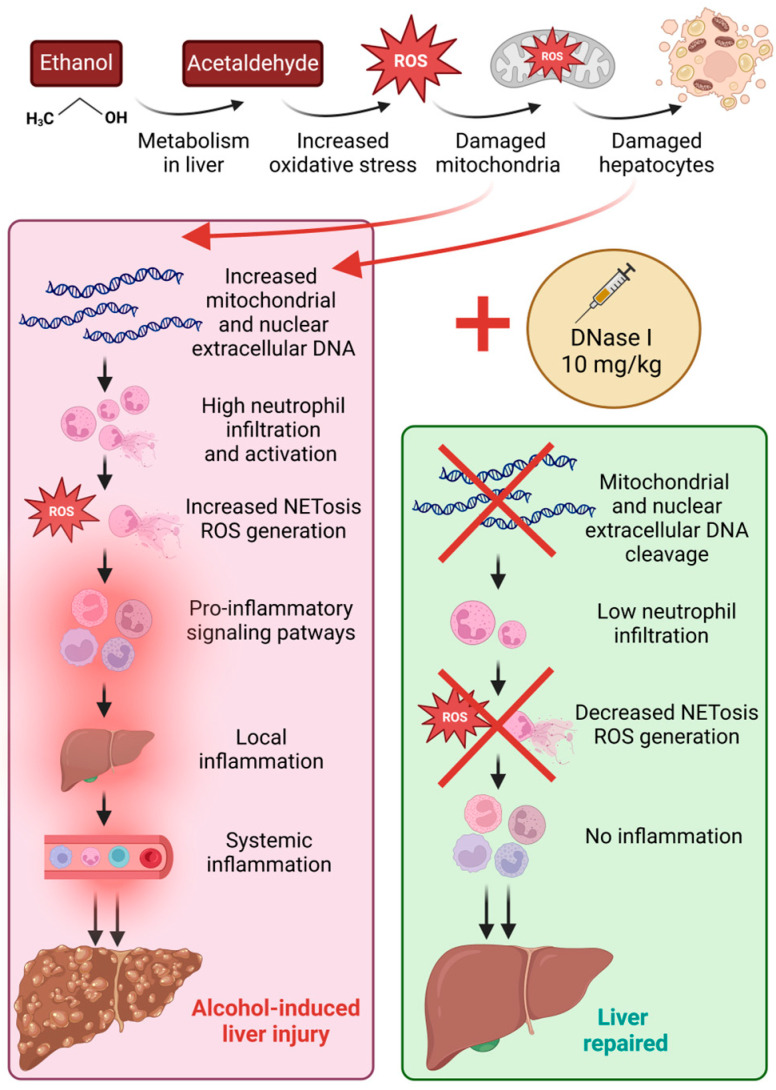
Proposed mechanisms of local and systemic inflammation in excessive alcohol consumption and ameliorative effect of DNase I treatment.

Recent research has highlighted the importance of circulating ecDNA and NETs-associated peripheral markers in MASLD that may play a crucial role in immune system activation and progression to MASH [12,13]. To our knowledge, these markers remain unexplored in ALD. Despite the fact that the initial stimuli inducing the inflammation may differ in these pathologies, the innate immune system, and specifically neutrophils, reacts in a similar manner. Therefore, our study aimed to investigate the role of these markers in ALD using the mouse model of chronic-plus-binge ethanol feeding. Simultaneously, we proposed and analyzed the effect of a novel treatment approach for ALD, DNase I, that could ameliorate liver tissue damage by decreasing the concentrations of immunogenic ecDNA and proinflammatory signaling pathways (Figure 5).

By the end of the experiment, a 23.5% mortality rate was reported in the EtOH group, but not in the control and DNase-treated mice, supporting the ameliorative outcome of DNase I treatment. Interestingly, other publications refer to no mortality after binge ethanol feeding; however, this could be a result of the age and sex differences of animals used across studies [19]. As expected, we detected a body weight decrease in experimental EtOH and EtOH + DNase groups over time, but without significant differences between individual groups. A similar trend of body weight decrease, within the first two days of the experiment, was described in the study protocol we followed [17]. Corresponding with the DNase I application from day 5, a body weight increase in the EtOH + DNase group was observed, indicating the positive effect of the applied treatment.

The activity of AST enzyme was not significantly higher in the ethanol-fed group compared to the control, although a clear trend is visible. One possible reason could be that the mouse model lasted only 10 days, compared to other chronic alcohol-induced rodent models with increased enzyme activities that usually span 4 weeks and more [20]. It has been reported that female rodents are generally more susceptible to ethanol-induced liver injury than males [17]. Therefore, we specifically used female mice to investigate neutrophil extracellular trap formation and ecDNA in a relatively short 10-day-long model. Surprisingly, female mice in our experimental settings could have been more resistant to ethanol feeding, as compared to studies using male mice [19]. Interestingly, the EtOH + DNase group showed lower AST activity, but higher ALT activity compared to the EtOH group without treatment. This finding indicates that DNase might have some direct effect on liver cells; however, the explanation is unclear. Nevertheless, liver histology clearly showed visible pathological changes after binge ethanol feeding.

Long-term excessive alcohol consumption leads to lymphocytopenia and functional disability of lymphocytes, increasing the risk of infections [21], as well as possibly inducing thrombocytopenia [22]. In the current experiment, chronic-plus-binge ethanol feeding led to a significant decrease in white blood cell, lymphocyte and platelet counts regardless of DNase I application. The observed reductions in the number of white blood cells, lymphocytes, and platelets in our study support the broad immunosuppressive and hematological impact of alcohol, consistent with the literature. The lack of improvement in these parameters with DNase I treatment indicates that DNase I does not directly mitigate the alcohol-induced changes in these specific cell types. It primarily targets ecDNA rather than directly influencing hematopoietic or immune cell recovery. Yet, we had expected that improvements in these parameters could happen indirectly via decreased inflammation and inhibited stimulation of the immune system by immunogenic ecDNA. A significant increase in neutrophils in the EtOH group was shown, similarly to another alcohol-induced liver injury study [23]. Additionally, a trend towards lower neutrophil counts in the EtOH + DNase group compared to controls was shown, yet not significant. According to the literature, an increased NLR ratio could be considered as another indicator of systemic inflammation [24]. Our study showed an increase in both NLR and PLR ratios in the EtOH group and showed a decreasing trend after DNase I treatment. Taken together, neutrophil count, NLR and PLR ratio results suggest that alcohol feeding promotes systemic inflammation and immune dysregulation. Despite the results not clearly demonstrating whether DNase I treatment significantly attenuated these increases, a clear trend is visible. In summary, alcohol feeding resulted in changes in immune cell profile, and DNase I application could potentially ameliorate these changes (Figure 5).

In ALD, neutrophils infiltrating the liver represent one aspect of innate immunity. Neutrophils, in addition to phagocytosis, also use NET formation in response to various sterile and infectious stimuli. Thus, they can act as both cells to aid in disease and as a source of sterile inflammation [25]. To investigate their role in systemic inflammation, we analyzed NET-associated peripheral markers. Concentrations of circulating total, ncDNA, mtDNA, and MPO-DNA complexes did not differ significantly across groups, although a clear trend towards higher levels of these markers is visible in the ethanol group versus the control. An increased ncDNA and mtDNA variability, compared to total ecDNA, could result from the different methods applied. A total ecDNA concentration considers short fragments that cannot be detected by qPCR. We hypothesized that increased mtDNA in the EtOH group could originate from dying hepatocytes, specifically from mitochondria damaged by increased oxidative stress and ROS production caused by ethanol feeding [3]. Based on previously published studies, plasma ecDNA concentrations of patients with ALD are higher compared with the control group [26]. As was shown, the majority of ecDNA that is released to the plasma of patients with liver damage and hepatocellular carcinoma originates from the liver [27,28]. Similarly, in the animal acute liver failure model, plasma ecDNA concentrations are higher and assumed to originate from the death of hepatocytes [29]. As expected, a chronic-plus-binge ethanol feeding accompanied by DNase I treatment increased serum DNase activity in the EtOH + DNase group in comparison to other groups, indicating an effective administration method and timing.

Despite increased neutrophil counts in the EtOH group, peripheral in vivo NETs formation did not change. This is in discrepancy with other studies that show higher NETs formation in alcohol-associated hepatitis [23]. Interestingly, a population of low-density neutrophils was found in mice with ALD. It was described as neutrophils with an exhausted phenotype, meaning they are less responsive to infections and show decreased phagocytosis [23]. Moreover, components of NETs, such as peptidyl arginine deiminase 4, were found to be associated with poor outcomes in patients with ALD [30]. On the contrary, other studies presented decreased NET formation due to alcohol, and in combined models with sepsis, this led to the decreased survival of mice and exacerbation of liver damage [31,32]. In this context, we analyzed the potential of neutrophils to form NETs under various stimuli ex vivo. As expected, peripheral ex vivo-induced NET formation was significantly increased after 3 h of incubation with bacterial (*E. coli*) or positive control (PMA) stimuli, compared to RPMI. However, there were no differences between neutrophils isolated from control mice and mice drinking ethanol. Interestingly, we noticed decreased PMA-induced ex vivo NET formation after chronic-plus-binge ethanol feeding regardless of DNAse I treatment. This corresponds with another study that also showed reduced NET formation in response to a PMA following alcohol binge drinking, and depletion of neutrophils in vivo reduces the inflammation [32]. In summary, our data show that chronic-plus-binge ethanol feeding does not induce the release of high amounts of ecDNA and NET formation in circulation, suggesting that chronic alcohol consumption induces systemic inflammation by a different mechanism. However, it may play a role in the ability of neutrophils to react to various stimuli and this can potentially increase susceptibility to infections in people with chronic alcohol consumption.

Lastly, we focused on local inflammation and neutrophil infiltration. The histology analysis clearly shows that chronic-plus-binge ethanol feeding resulted in elevated ALD histological score, represented primarily by steatosis, as well as elevated fibrosis and lobular infiltrations of neutrophils. All these three aspects showed a significant decrease or an improved score trend after DNAse I treatment, indicating the ameliorative effect of its application. Neutrophil infiltration in the liver has been reported in numerous studies [33,34,35], but the effect of DNase I treatment in ALD has not been previously described.

The novelty of this study lies in several key aspects. Firstly, the study analyzes circulating ecDNA and NETs-associated peripheral markers in the context of ALD, which, to our knowledge, have not been studied before in this pathology. Secondly, the study investigates the use of DNase I as a novel therapeutic approach for ALD, demonstrating its potential to ameliorate liver damage by improving markers of systemic inflammation, liver histology, and immune cell profile. Based on the available clinical studies, DNase I is considered a non-harmful drug with no effect on reproduction in animal studies as well as no toxicity detected. It has been used as a treatment in clinical trials for sepsis, cystic fibrosis, and COVID-19, via inhalation or intravenous injection [36,37,38]. Lastly, the study’s findings highlight the histological improvements in neutrophil infiltration scores and fibrosis with DNase I treatment, presenting new therapeutic possibilities in ALD.

The limitation of this study is the duration of the experiment. The lack of significant increase in systemic NET formation could be due to the relatively short timeframe of the model. During early alcohol exposure, compensatory mechanisms, such as antioxidant responses or anti-inflammatory pathways, might suppress excessive systemic NET formation. Additionally, DNase I was administered for only five days, which may not fully capture its long-term effects. Given the short half-life of DNase I efficacy, the administration must be repeated every 12 h, making the treatment relatively costly. Subsequently, this affected the EtOH + DNase group sample size. Future research should explore extended treatment durations, such as a one-month model, which has been previously established using the Lieber–DeCarli ethanol diet [20].

## 4. Materials and Methods

### 4.1. Mice and Housing Conditions

All animal experiments were conducted on adult 6-month-old female C57BL/6J mice (C57BL/6J, Strain #000664, Jackson Laboratory, Charles River, Germany). Animals were housed in polyethylene cages (36.5 cm × 20.5 cm × 14 cm) and kept under constant conditions as follows: 55 ± 10% humidity, 24 ± 2 °C temperature, and a 12 h long light/dark cycle.

### 4.2. Experiment Design

The experimental design and diet preparation followed the protocol of Bertola et al. [17]. Animals were randomly assigned into three groups: the control group (CTRL; n = 13), the experimental ALD group (EtOH; n = 17) and the experimental ALD group with DNase I treatment (EtOH + DNase; n = 5). Both ALD groups received the Lieber–DeCarli alcohol liquid diet (Bio-Serv, Frenchtown, NJ, USA) containing 5% ethanol, while the CTRL group was fed an isocaloric control liquid Lieber–DeCarli diet (Bio-Serv, Frenchtown, NJ, USA) without ethanol. DNase I (10 mg/kg; Sigma-Aldrich, Steindheim, Germany) was administered to EtOH + DNase mice intraperitoneally every 12 h from day 5 until the end of the experiment. All groups had ad libitum access to their respective liquid diets, with no additional water source for 10 days. Before the experiment, all groups were acclimatized to liquid feeding by being provided the control Lieber–DeCarli diet ad libitum for 5 days. During the experiment, body weight was monitored every other day. On day 11, ALD groups received a single binge dose of ethanol (5 g/kg body weight) by oral gavage method. The CTRL group received an equivalent volume of saline. Animals were euthanized 9 h after binge.

### 4.3. Blood and Organ Collection

At the end of the experiment, all animals underwent cardiac perfusion and cervical dislocation. Animals were anesthetized using 3% isoflurane inhalation anesthesia (1000 mg/g, Vetpharma Animal Health, Barcelona, Spain). Blood was collected from the retro-orbital plexus into anticoagulant EDTA collection tubes (Sarstedt AG & Co. KG, Nümbrecht, Germany) and 1.5 mL Eppendorf tubes, for plasma and serum analyses, respectively. The blood count was analyzed using the hemoanalyzer Abacus VET 5 (Diatron MI ZRT., Budapest, Hungary). The liver samples were weighed and sectioned for histology analysis. All procedures were performed to minimize animal suffering.

### 4.4. Quantification of NET Formation

Four 30 µL aliquots of whole blood were prepared to assess the percentage of neutrophils producing NET formation. The first aliquot represented the state of NET formation in whole blood, the remaining three aliquots were incubated for 3 h at 37 °C with distinct stimuli to induce NET formation: (1) 10 µL of RMPI 1640 Medium (P04-16500, PAN-Biotech, Aidenbach, Germany) supplemented with 10% FBS (P30-3306, PAN-Biotech, Aidenbach, Germany); (2) 3 µL of *Escherichia coli* CFT073 (*E. coli*; ATCC 700928; https://www.atcc.org/products/700928) from an overnight culture with optical density 1 equaled 8 × 10^8^/mL of colony forming units; (3) 1.2 µL of 40 µM stock of phorbol-12-myristate-13-acetate (PMA) (P8139-1MG, Saint-Louis, MO, USA). The erythrocytes were lysed using an ice-cold isotonic ammonium chloride buffer, consisting of 150 mM NH4Cl, 10 mM KHCO3 and 0.1 mM EDTA for 30 min on ice. Subsequently, leukocytes were centrifuged at 400× *g* for 10 min at 4 °C, and the pellet was resuspended in 100 µL of antibody cocktail containing FACS buffer (RPMI + 1% FBS), 2.5 µg/sample of Alexa Fluor^®^ 647 anti-mouse Ly-6G antibody (127610, Biolegend, San Diego, CA, USA), 0.1 µg/sample of primary rabbit monoclonal anti-Histone H3 (citrulline R17) antibody (ab219407, Abcam, Cambridge, UK), 0.025 µg/sample of secondary Brilliant Violet 510™ Donkey anti-rabbit IgG antibody (406419, Biolegend) and 200 nM SYTOX™ Green Nucleic Acid Stain (S7020, Invitrogen, Eugene, OR, USA) for 15 min at room temperature in the dark and subsequently measured using a DxFlex flow cytometer (Beckman Coulter, Brea, CA, USA). Data were analyzed using FCSExpress v. 6.06.0040 software (De Novo Software, Pasadena, CA, USA). Percentages of NET formation were identified by Ly-6G positivity with co-expression of extracellular DNA and citrullinated histone H3, and their counts were normalized per 1 milliliter of blood.

### 4.5. Liver Damage Markers

Plasma samples were obtained by centrifugation at 1600× *g* for 10 min at 4 °C. Liver enzyme activities, plasma AST and ALT (Merck & Co., Rahway, NJ, USA) were analyzed with the commercially available assay kits according to the manufacturer’s protocols.

### 4.6. Quantification of Myeloperoxidase—DNA Complexes

Plasma was obtained in the same manner as described above. The presence of MPO-DNA complexes was assessed using a modified ELISA protocol [39,40]. Briefly, a 96-well plate (Sarstedt, Nümbrecht, Germany) was coated overnight at 4 °C with 0.5 µg/mL anti-MPO antibody (0400-0002, Bio-Rad Laboratories, Hercules, CA, USA) in 0.1 M carbonate–bicarbonate buffer. Bovine serum albumin (Merck, Saint-Louis, MO, USA) in PBS (BR0095, Canvax Reagents SL, Valladolid, Spain) was used for blocking and sample dilution, and 100-times-diluted Anti-DNA POD antibody (11774425001, Roche, Basel, Switzerland) was used.

### 4.7. Extraction and Quantification of Extracellular DNA

For ecDNA extraction, plasma was centrifuged in the same manner as described above, followed by the second centrifugation at 16,000× *g* at 4 °C for 10 min. Samples were extracted with the QiaAmp DNA Blood Mini Kit (QIAGEN GmbH, Hilden, Germany) with minor modifications: initial sample volume of 100 µL and elution volume of 50 µL. The concentration of extracted ecDNA was determined using a Qubit dsDNA HS assay kit (Thermo Fisher Scientific, Waltham, MA, USA) and Qubit 3.0 fluorometer (Thermo Fisher Scientific, Waltham, MA, USA).

### 4.8. Quantification of Nuclear and Mitochondrial ecDNA

A real-time PCR method was used for analyzing the subcellular origin of ecDNA, specifically the concentration of nuclear-specific (ncDNA) and mitochondrial-specific (mtDNA) ecDNA. The PCR reaction mix was composed of SsoAdvanced Universal IT SYBRGreen SuperMix (Bio-Rad Laboratories, Hercules, CA, USA) and 250 nM each of forward and reverse primers. For ncDNA analysis, forward 5′-TGT CAG ATA TGT CCT TCA GCA AGG-3′ and reverse 5′-TGC TTA ACT CTG CAG GCG TAT G-3′ were used. For mtDNA, the primers were as follows: forward 5′-CCC AGC TAC TAC CAT CAT TCA AGT-3′ and reverse 5′-GAT GGT TTG GGA GAT TGG TTG ATG T-3′. A real-time thermal cycler qTOWER^3^ Series (Analytik Jena GmbH + Co, Jena, Germany) was used with the following cycle settings: an initial denaturation step at 95 °C for 15 min, 40 cycles of denaturation at 94 °C for 15 s, annealing at 60 °C for 30 s, and polymerization at 72 °C for 30 s. For the melting curve, an additional annealing step at 65 °C for 15 s was performed. The quantifications of ncDNA and mtDNA were measured as gene equivalents (GEs) per milliliter (mL) of plasma.

### 4.9. Deoxyribonuclease Activity

A single radial enzyme dispersion (SRED) assay was applied for nonspecific deoxyribonuclease I (DNase I) activity determination. At first, serum samples were obtained by centrifugation at 1600× *g* at 4 °C for 10 min. Samples were loaded onto a 1% agarose gel. The gel consisted of 0.5 mg/mL DNA isolated from chicken livers, green-fluorescent dye GoodView™ (SBS Genetech, Beijing, China), 10 mM MgCl2, 10 mM CaCl2, and 0.5 M Tris–HCl with a final pH of 7.5. For calibration, dilutions of DNase I (QIAGEN GmbH, Hilden, Germany) were used. The gel was incubated in the dark at 37 °C for 17 h. The diameter of the dark circle of hydrolyzed DNA, correlating linearly with the amount of enzyme, was measured using ImageJ software v. 1.53e (NIH Image, Bethesda, MD, USA).

### 4.10. Liver Tissue Histology

Liver tissue samples from experimental animals were collected and fixed in 4% formaldehyde for 48 h, routinely processed by embedding in paraffin, cut in 4 μm thick slices, stained with hematoxylin and eosin (H&E) and evaluated by light microscopy (Leica DM2000, Wetzlar, Germany). Each sample was evaluated for the presence of steatosis (score 0–3), hepatocyte ballooning (score 0–2), and lobular inflammation (score 0–3). The ALD histological score was calculated as the total sum of all three categories [41]. Staining according to Van Gieson was performed for evaluation of collagen tissue proportion in the liver tissue. Data were analyzed as fibrosis scores [41]. Naphthol-AS-D-chloroacetate esterase (CHAE) activity served for the detection of tissue infiltration by granulocytes.

### 4.11. Ethical Statement

All procedures were approved by the Ethical Committee of the Institute of Pathophysiology, Comenius University, Bratislava, Slovakia (approval no. 07/2023, 4503-3/2024-220), following the EU Directive 2010/63/EU and Slovak legislation.

### 4.12. Statistical Analysis and Calculations

Statistical data analysis and graph visualization were performed using GraphPad Prism version 8.0.1 (GraphPad Software, Inc., San Diego, CA, USA). Differences across groups were analyzed with a one-way ANOVA and Tukey’s multiple comparison test. Two-way ANOVA with repeated measures and post hoc Bonferroni corrections were applied for dynamics analysis. Statistical *p*-values less than 0.05 were considered significant.

## 5. Conclusions

Here, we analyzed the role of neutrophils in systemic and local inflammation induced by a mouse model of chronic and binge ethanol feeding. Based on the results, we assume that the chronic consumption of ethanol probably does not result in systemic but rather local NETosis-associated processes, presumably from the application of a short-duration model. This study showed for the first time that administration of DNase I has strong therapeutic potential in ALD. However, more research is needed to prove its positive effect on local liver-targeted inflammation.

## Data Availability

Data contained within the article.
